# Overexpression of miRNA-25-3p inhibits Notch1 signaling and TGF-β-induced collagen expression in hepatic stellate cells

**DOI:** 10.1038/s41598-019-44865-1

**Published:** 2019-06-12

**Authors:** Berit Genz, Miranda A. Coleman, Katharine M. Irvine, Jamie R. Kutasovic, Mariska Miranda, Francis D. Gratte, Janina E. E. Tirnitz-Parker, John. K. Olynyk, Diego A. Calvopina, Anna Weis, Nicole Cloonan, Harley Robinson, Michelle M. Hill, Fares Al-Ejeh, Grant A. Ramm

**Affiliations:** 1 Hepatic Fibrosis Group, QIMR Berghofer Medical Research Institute, Brisbane, Queensland Australia; 2 Mater Research, Translational Research Institute, Brisbane, Queensland Australia; 3 Faculty of Medicine, The University of Queensland, Brisbane, Queensland Australia; 4 Personalised Medicine Team, QIMR-Berghofer Medical Research Institute, Brisbane, Queensland Australia; 5 School of Pharmacy and Biomedical Sciences, Curtin Health Innovation Research Institute, Curtin University, Bentley, WA Australia; 6 School of Veterinary and Life Sciences, Murdoch University, Perth, Western Australia Australia; 7 Department of Gastroenterology & Hepatology, Fiona Stanley Fremantle Hospital Group, Murdoch, Western Australia Australia; 8 School of Medical and Health Sciences, Edith Cowan University, Joondalup, Western Australia Australia; 9 Genomic Biology Lab, QIMR-Berghofer Medical Research Institute, Brisbane, Queensland Australia; 10 Precision & Systems Biomedicine, QIMR-Berghofer Medical Research Institute, Brisbane, Queensland Australia

**Keywords:** Mechanisms of disease, Liver fibrosis

## Abstract

During chronic liver injury hepatic stellate cells (HSCs), the principal source of extracellular matrix in the fibrotic liver, transdifferentiate into pro-fibrotic myofibroblast-like cells - a process potentially regulated by microRNAs (miRNAs). Recently, we found serum miRNA-25-3p (miR-25) levels were upregulated in children with Cystic Fibrosis (CF) without liver disease, compared to children with CF-associated liver disease and healthy individuals. Here we examine the role of miR-25 in HSC biology. MiR-25 was detected in the human HSC cell line LX-2 and in primary murine HSCs, and increased with culture-induced activation. Transient overexpression of miR-25 inhibited TGF-β and its type 1 receptor (TGFBR1) mRNA expression, TGF-β-induced Smad2 phosphorylation and subsequent collagen1α1 induction in LX-2 cells. Pull-down experiments with biotinylated miR-25 revealed Notch signaling (co-)activators ADAM-17 and FKBP14 as miR-25 targets in HSCs. NanoString analysis confirmed miR-25 regulation of Notch- and Wnt-signaling pathways. Expression of Notch signaling pathway components and endogenous Notch1 signaling was downregulated in miR-25 overexpressing LX-2 cells, as were components of Wnt signaling such as Wnt5a. We propose that miR-25 acts as a negative feedback anti-fibrotic control during HSC activation by reducing the reactivity of HSCs to TGF-β-induced collagen expression and modulating the cross-talk between Notch, Wnt and TGF-β signaling.

## Introduction

Hepatic stellate cells (HSCs) are one of the major players in fibrotic processes associated with wound healing in chronic liver injury. In the healthy organ they are part of the non-parenchymal cell population occupying the space of Disse between hepatocytes and sinusoidal endothelial cells. Despite their well-described role as vitamin A storing cells, HSCs are also involved in the regulation of sinusoidal blood pressure, immune cell modulation and liver homoeostasis^1^. Upon activation resulting from tissue damage, inflammation and concomitant soluble mediators such as transforming growth factor beta (TGF-β), HSCs transdifferentiate into myofibroblast-like cells exhibiting proliferative, contractile and fibrogenic properties, becoming the major source of fibrillary collagens (I and III) in the fibrotic liver^2^. HSCs also engage in complex cross-talk with adjacent cells to promote fibrosis progression. As part of their phenotypic morphogenesis during activation, fundamental changes in gene expression patterns and intracellular signaling pathways occur^3–6^. One of the most studied pathways is the TGF-β signaling pathway which acts as an autocrine feedback loop to stimulate collagen expression in activated HSCs^7^. Components of the Wnt and Notch pathways are also differentially expressed in activated compared to quiescent HSCs^8,9^ and cross-talk between both pathways is emerging as a key regulatory mechanism in liver fibrogenesis^10,11^. However, the mechanisms coordinating the interaction between the different signaling pathways regulating HSC activation have not been examined in great detail. In this regard, the recent emergence of microRNAs, which are able to fine-tune gene expression, raises a novel approach to investigate and manipulate mechanisms of HSC activation^12^.

MicroRNAs (miRNAs) are 18–24 nucleotide RNAs that modulate gene expression by targeting specific mRNAs via complementary base-pair binding. Canonically, miRNAs interact with the 3′ untranslated region (UTR) of their target mRNAs in cytoplasmic RNA-induced silencing complexes (RISC) to repress translation and/or induce degradation^13^. MiRNA can also induce gene expression by binding outside the 3′UTR^14^. Importantly, miRNAs not only affect their cell of origin, they can also be transported via extracellular vesicles to other target cells within the body, acting via paracrine and endocrine cell-cell-communication processes^15,16^. With over 2500 human miRNAs described^17^, and up to hundreds of potential mRNA targets each^18^, miRNAs have been implicated in a number of different molecular and cellular pathways of chronic liver disease pathobiology. Specifically, several miRNAs, e.g. miR-122 and miR-21 have been shown to play a role in the development of liver fibrosis, regulating processes including tissue inflammation, cell apoptosis, as well as activation of HSCs^19^. Moreover, due to a disease-dependent release into the blood stream and their relative stability in serum, miRNAs are potential non-invasive biomarkers for the detection of liver disease severity and carcinogenesis^20^.

We have recently demonstrated an increased abundance of miRNA-25-3p (miR-25) in serum of children with cystic fibrosis (CF) who have no liver disease involvement, compared to both CF children with liver disease and healthy individuals^21^. This led us to propose that miR-25 may play a role in regulating the development of liver disease, via repression of pro-fibrogenic pathways in HSCs and other liver cells. Although miR-25 expression in liver tissue has mainly been investigated in the context of cancer, where it has been shown to tissue-dependently promote or inhibit cancer development^22,23^, there is evidence for anti-fibrotic effects in other organs. Caskey and colleagues described a negative correlation between miR-25 and collagen I expression in a skin injury model in diabetic mice^24^, and downregulation of collagen expression by transfection with miR-25 was shown in cardiac fibroblasts^25^.

In the current study we investigated the expression of miR-25 during HSC activation and its impact on activation-related genes and signaling pathways. We demonstrated that miR-25 overexpression in HSCs diminishes expression of the sheddase A Disintegrin And Metalloproteinase 17 (ADAM-17) and the γ-secretase co-activator FK506 Binding Protein 14 (FKBP14), leading to inhibition of downstream Notch, TGF-β and Wnt signaling. As a result, HSCs become less sensitive to TGF-β stimulation, with resultant decreased collagen expression. At the same time miR-25 was upregulated in primary HSCs after culture-induced and *in vivo* activation and in liver tissue of mice with hepatic fibrosis. Therefore, we suggest that miR-25 expression is part of a negative feedback loop during liver fibrosis that dampens the responsiveness of HSCs to persistent fibrotic stimuli and therefore mitigates excessive collagen secretion.

## Results

### MiR-25 overexpression decreases TGF-β signaling in the human HSC cell line LX-2

**Figure 1:**
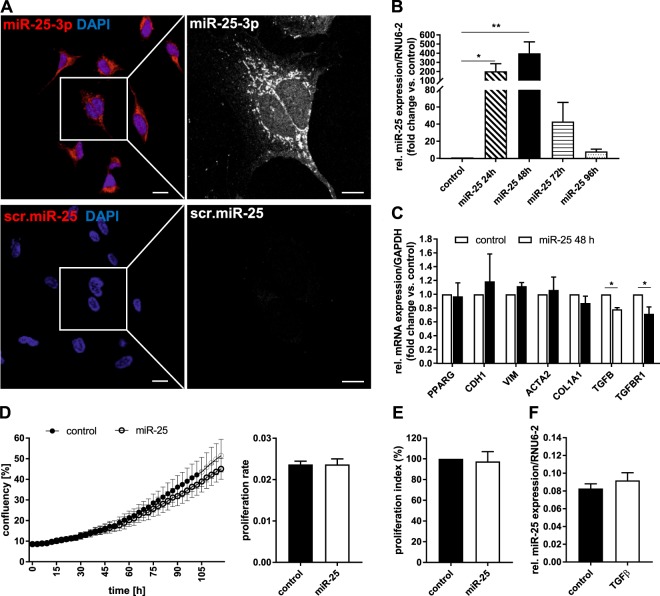
*In vitro* analysis of the effect of miR-25 overexpression on the activation status of human hepatic stellate cells (HSCs). (**A**) *In situ* hybridization of LX-2 cell line with DIG-labeled miR-25 specific probes (red). A scrambled miR-25 probe was used as negative control (scale bar: left panel 100 µm, right panel 10 µm). (**B**) Relative quantification of miR-25 expression 24, 48, 72 and 96 h after transfection of LX-2 cells with miR-25 mimics (n = 3–4). (**C**) Analysis of relative mRNA expression of different HSC marker for quiescence (PPAR-γ (PPARG), E-cadherin (CDH1)) and activation (vimentin (VIM), αSMA (ACTA2), collagen 1a1 (COL1a1), TGF-β1 (TGFB)) as well as TGF-β receptor type 1 (TGFBR1) in miR-25 over-expressing compared to control miR transfected cells 48 h after transfection (n = 5–7). Proliferation analysis using Incucyte confluency measurement (n = 3) (**D**) or MTT assay (n = 5) (**E**) in control and miR-25 over-expressing LX-2 cells. (**F**) Relative quantification of miR-25 expression in untreated and TGF-β treated (5 ng/ml for 24 h) LX-2 cells (n = 5). (*p < 0.05 vs control).

To investigate the endogenous expression of miR-25 and its localization in human HSCs, we performed fluorescence *in-situ* hybridization (FISH) experiments in the activated human HSC cell line, LX-2 (Fig. 1A). Confocal microscopy showed strong punctate staining for miR-25 in the cytoplasm, possibly corresponding to RISC complexes, as well as diffuse staining in the nuclei (Fig. 1A upper panels). The control probe, comprising a scrambled miR-25 sequence, revealed no positive staining (Fig. 1A, lower panels).

**Figure 2:**
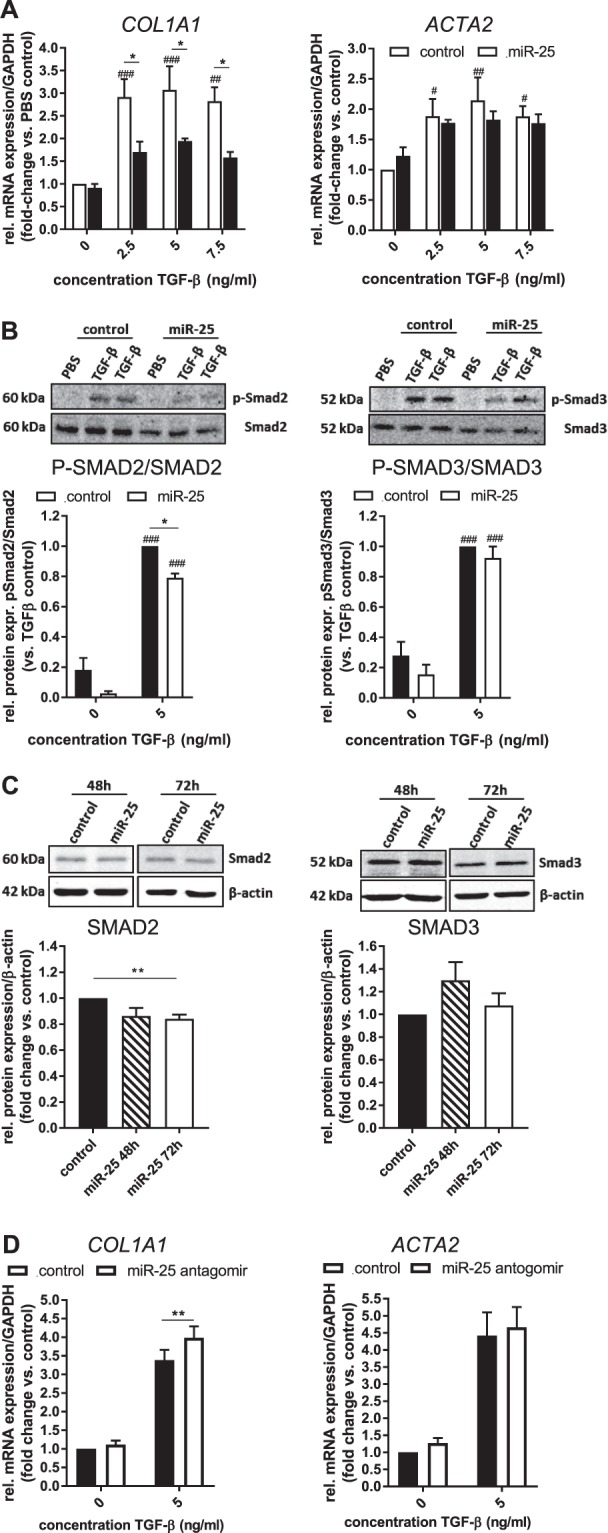
*In vitro* analysis of the effect of miR-25 overexpression on TGF-β signaling in LX-2 cells. (**A**) qRT-PCR analysis of collagen1a1 (*COL1A1*) and αSMA (*ACTA2*) mRNA expression (n = 3) and (**B**) Western Blot analysis of phosphorylation of SMAD2 (n = 4) and SMAD3 (n = 8) proteins after TGF-β stimulation (5 ng/ml for 24 h (mRNA) and 6 h (protein)) in miR-25- or control-transfected LX-2 cells. (**C**) Western Blot analysis of TGF-β signaling components SMAD2 and SMAD3 in miR-25 overexpressing LX-2 cells compared to control cells (n = 7–8). Cropped Western Blot images originate from the same (target vs. loading control) or different membranes (phosphorylates vs. whole protein; time points). (**D**) qRT-PCR analysis of collagen1a1 (*COL1A1*) and αSMA (*ACTA2*) mRNA expression after TGF-β stimulation (5 ng/ml for 24 h) in miR-25 antagomir- (inhibitor) or control antagomir-transfected LX-2 cells. (*p < 0.05 vs control; ^#^p < 0.05 vs. PBS; ^##^p < 0.01 vs. PBS; ^###^p < 0.001 vs. PBS).

To examine the function of miR-25, we conducted transient overexpression of miR-25 mimic (small RNA duplexes that imitate the mature miRNA molecule) in LX-2 cells. We were able to further increase the endogenous miRNA expression up to 400-fold (48 h after transfection) compared to control-transfected samples at the same time point (Fig. 1B). This increased expression of exogenous miR-25 was evident up to 96 h after a single transfection with a marked decrease after 48 h (Fig. 1B). Overexpression of miR-25 had no significant effect on the expression of HSC quiescence (Peroxisome proliferator-activated receptor gamma (PPAR-γ) [*PPARG*], E-cadherin [*CDH1*]) or activation (vimentin [*VIM*], alpha smooth muscle actin (αSMA) [*ACTA2*], collagen 1a1 [*COL1A1*]) markers examined by qRT-PCR when compared to LX-2 cells transfected with control miR (Fig. 1C), nor on the proliferation rate of the cells, as assessed by growth rate (confluency) measurements (Fig. 1D) and metabolic activity (MTT assay) (Fig. 1E). However, the mRNA expression of TGF-β and its type 1 receptor [*TGFBR1*] were significantly reduced in miR-25 overexpressing LX-2 cells (Fig. 1C). Treatment with the HSC activator TGF-β (5 ng/ml) for 24 h did not change miR-25 expression in LX-2 cells (Fig. 1F). To assess whether the downregulation of transforming growth factor beta receptor type 1 (TGF-βRI) has a functional impact on TGF-β-induced HSC activation, we stimulated LX-2 cells with increasing concentrations of recombinant TGF-β1 and measured the expression of the HSC activation marker *ACTA2* and *COL1A1* mRNA by qRT-PCR. MiR-25 overexpression resulted in an inhibition of TGF-β-induced collagen 1a1 (*COL1A1*) expression (Two-way ANOVA, p < 0.0001), whereas TGF-β-induced αSMA (*ACTA2*) mRNA upregulation was not affected (Fig. 2A). We further investigated the potential inhibitory mechanism of miR-25 on TGF-β signaling by assessing TGF-β-induced phosphorylation of Mothers against decapentaplegic homolog 2 (SMAD2) and 3 proteins by Western blot analysis, both downstream signaling molecules of the TGF-β receptor. The ratio of phosphorylated SMAD2 (P-SMAD2) to total SMAD2 expression was significantly reduced after miR-25 overexpression compared to control miR-transfected LX-2 post TGF-β stimulation (Fig. 2B), while the phosphorylation of SMAD3 was not significantly decreased by miR-25. Furthermore, miR-25 overexpression in LX-2 cells resulted in a decreased protein expression of SMAD2, whereas there was no significant effect on SMAD3 expression (Fig. 2C). Additionally, we analysed the effect of miR-25 inhibition using a miR25 antagomir in TGF-β-stimulated LX-2 cells revealing a significantly increased TGF-β-induced mRNA expression of *COL1A1*, but not αSMA (*ACTA2*) in cells treated with miR-25 antagomir compared to control antagomir-treated cells (Fig. 2D). Taken together, these results show that miR-25 overexpression reduced the sensitivity of human HSCs to TGF-β stimulation by downregulating TGF-βRI and the subsequent inhibition of downstream signaling via SMAD2.

### Biotin-pull-down assay reveals components of the Notch signaling pathway as targets of miR-25

**Figure 3:**
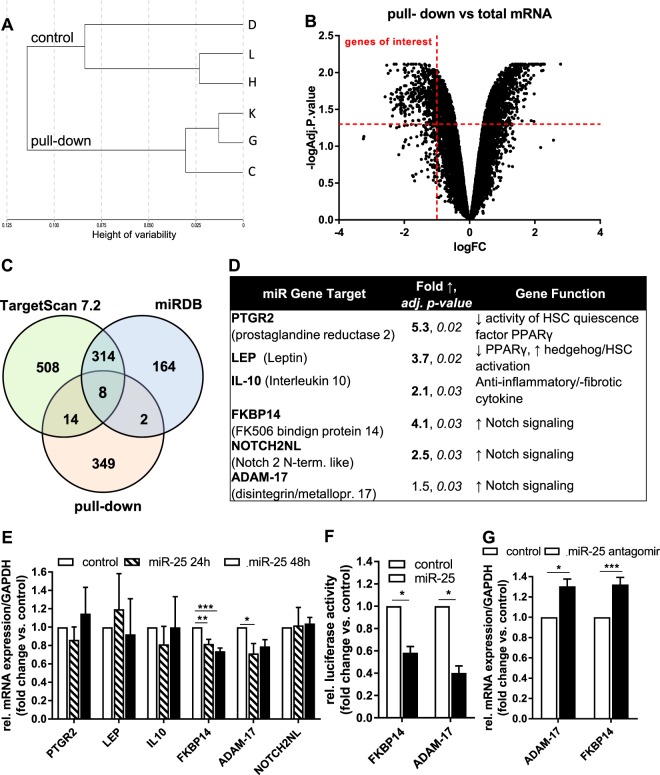
Identification of miR-25 biologically relevant targets using biotin pull-down assay. (**A**) Hierarchical clustering of Illumina sequencing data using the lumi package to demonstrate sample relationship between whole mRNA lysates (control, samples D, H, L) and enriched miRNA target mRNA lysates (pull-down, samples C, G, K). (**B**) Volcano plot depicting log2-transformed fold-change against log10-transformed adjusted p-value of genes detected above background level of the array in pull-down compared to whole cell lysate samples (fold change ≤−2; adj. p-value < 0.05). (**C**) Venn diagram showing the overlap between miR-25 predicted targets by TargetScan, miRDB and biotin pull-down assay. (**D**) Selected Putative miR-25 target mRNA enriched by biotin-pulldown assay (**E**) qRT-PCR expression analysis of selected predicted target mRNA regulated by miR-25 (n = 4–6). (**F**) Validation of miR-25 binding to predicted target mRNA by luciferase-assay (n = 6–7). (**G**) Analysis of relative mRNA expression of target genes ADAM-17 and FKBP14 in LX-2 cells treated with miR-25 antagomir (inhibitor) or control antagomir (n = 9–10). (*p < 0.05 vs control; **p < 0.01 vs. control; ***p < 0.001 vs. control).

Little is known of the role of miR-25 in the liver and no HSC-specific miR-25 mRNA targets have been previously described. Thus, we performed a biotin-based pull-down assay in LX-2 cells to uncover biologically relevant miR-25 targets in HSC. In this experimental setting, target mRNAs bound to a biotinylated miR-25 duplex transfected into LX-2 cells were captured using streptavidin-coated beads. The captured mRNA molecules were then surveyed using microarrays to identify targets that were significantly enriched in pull-down compared to whole cell lysate samples. Whole cell lysate samples were extracted from the transfected cells immediately before starting the pull-down to control for changes that might occur due to the transfection process alone and are unlikely to be identified by control cells transfected with scrambled miRNA. Hierarchical clustering of the sequencing data set (Fig. 3A) allowed a distinction between pull-down and control (whole cell lysate) samples, showing a high similarity of samples inside each group. During processing of the microarray data, low detection signal from the probes in one of the control sample was noticed and therefore this sample was excluded from further analysis. In the remaining samples, we identified 373 putative target mRNAs with a fold change of ≥2 and an adjusted p-value of < 0.05 (Fig. 3B) (Supplementary Data 1). Comparing the pull-down enriched putative targets with miR-25 predicted targets from TargetScan and miRDB miRNA target prediction tools, we observed an overlap of 8 genes (Fig. 3C, Supplementary Data 1). Interestingly, neither TGF-β nor TGF-βRI were among the significantly enriched mRNAs in the pull-down samples or any of the target prediction tools, suggesting that miR-25 indirectly down-regulates their expression. We did not choose to assess the 8 overlapping genes in the target prediction tools further, rather we selected 6 putative miR-25 targets out of the genes identified by the pull-down experiments, as they were genes that had the potential to regulate HSC activation (Fig. 3D). These include Notch pathway components (Notch 2 N-terminal like [NOTCH2NL], FKBP14), PTGR2 (Prostaglandin reductase 2, implicated in inhibiting the HSC quiescence marker PPARγ), IL10 (Interleukin 10, an anti-fibrotic cytokine) and LEP (leptin, a stimulator of HSC activation). Additionally, we included the Notch signaling regulator ADAM-17 in our analysis, as it was also a potential miR-25 target gene (Fig. 3D). As shown in Fig. 3E, qRT-PCR analysis revealed 2 out of 6 mRNAs were significantly downregulated in LX-2 cells after transient transfection with miR-25. These 2 putative targets (ADAM-17 and FKBP14) were further validated as targets of miR-25 via luciferase-miRNA target reporter assay (Fig. 3F). Accordingly, inhibition of miR-25 expression using a miR-25 antagomir increased mRNA expression of both ADAM-17 and FKBP14 in LX-2 cells (Fig. 3G). Hence, we identified ADAM-17 and FKBP14, components of the Notch signaling pathway, as biologically relevant targets of miR-25 in HSCs.

### Notch and Wnt signaling components are differentially expressed in miR-25 overexpressing LX-2 cells

**Figure 4:**
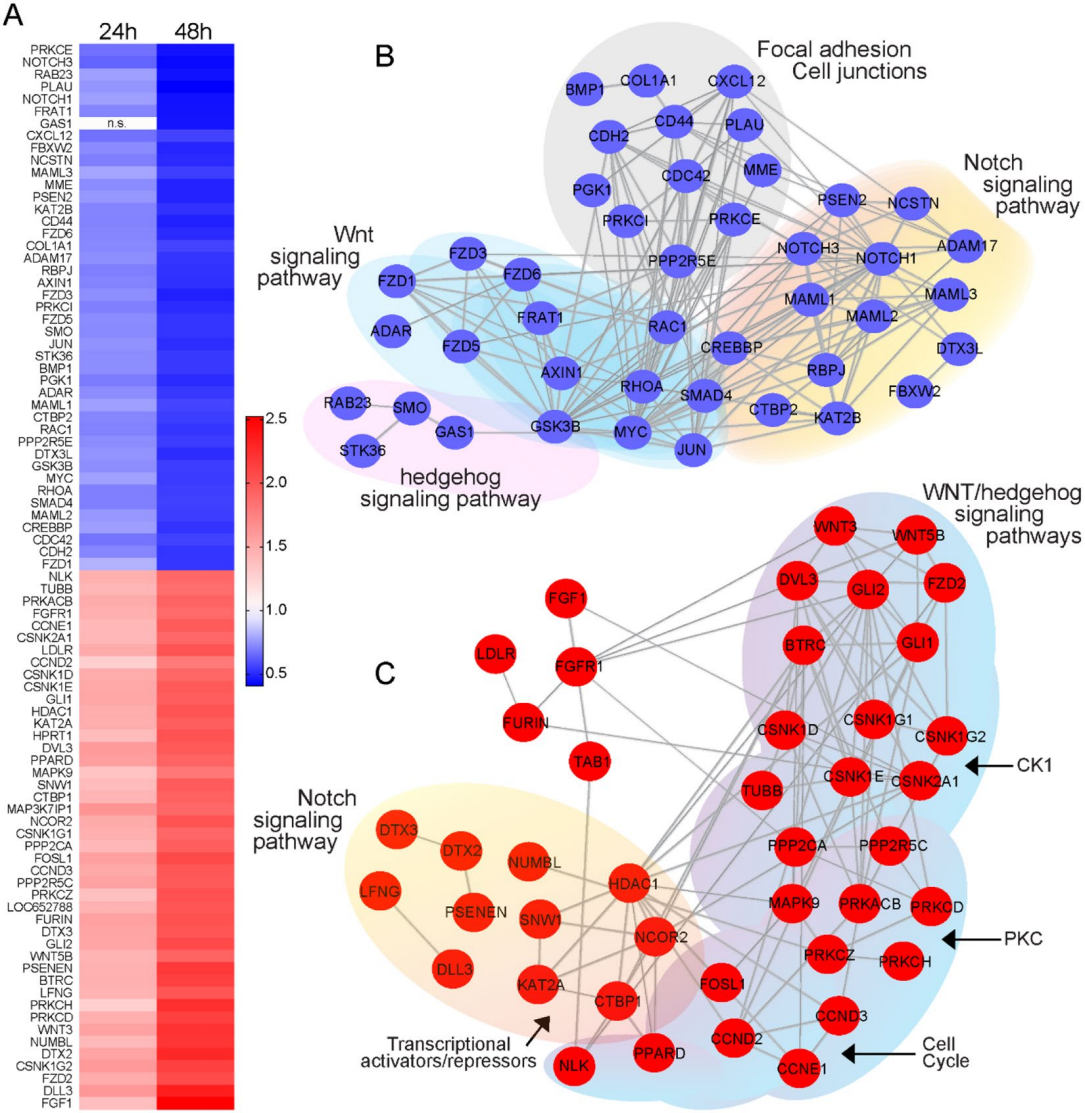
NanoString mRNA analysis of stem cell-related signaling pathways in miR-25 overexpressing LX-2 cells. (**A**) Heat map of the mRNA expression ratio of the measured genes in miR-25 vs. control transfected LX-2 cells 24 and 48 h after transfection. Each column represents the average of triplicates per time point (data for each replicate is in Supplementary Data 2). Statistical evaluation was carried out with two-way ANOVA corrected for multiple comparisons by false discovery rate (FDR) using two-stage linear step-up procedure of Benjamini, Krieger and Yekutieli (GraphPad® Prism). The q values (FDR) and individual *P* values are shown in Supplementary Data 2. The genes shown in the heatmap were statistically different (q < 0.05) between miR-25-transfected cells and control-transfected cells; non-statistically significant differences are marked as n.s. Genes downregulated in miR-25 overexpressing cells are presented in blue, upregulated genes in red. Colour scale represents fold change. String pathway analysis of genes downregulated (**B**) and upregulated (**C**) in miR-25 transfected LX-2 cells compared to control cells.

As both validated miR-25 targets act on the Notch pathway, next we analysed the changes in LX-2 cell mRNA expression, focussing on the Notch and Wnt signaling pathways at 24 and 48 h post miR-25 transfection. For this experiment, we used the NanoString mRNA Stem Cell Panel, which includes 199 genes involved in pluripotency and differentiation (including Notch, Wnt, and Hedgehog signaling components) (Supplementary Data 2). Of the 199 genes in the panel, 46 genes were not expressed above background, 27 genes were not expressed in all replicates (n = 3 per time point, 24 or 48 h post transfection), 28 genes were not affected by miR-25 expression, and 11 genes were only affected in one out of the three replicates (Supplementary Data 2). The remaining 87 genes (43.7%) were dysregulated by miR-25 overexpression compared to control transfection (fold change >1.5; false discovery rate q <0.05). As shown in Fig. 4A and Supplementary Data 2, ~50% of the genes were downregulated (<−1.5-fold) while the remainder were up-regulated (>1.5-fold), after correction for transfection efficiency. We used String-database analysis to visualize the protein-protein interactions of the molecules and pathways affected by miR-25 overexpression. Since we used a targeted panel for expression profiling, it was not appropriate to report pathway enrichment analysis. The downregulated genes (Fig. 4B) included genes belonging to the Notch (e.g. *NOTCH1*, *NOTCH*3, *ADAM-17*, *RBPJ*), Wnt (*FZD1*, *3*, 5 and 6, *AXIN1*) and Hedgehog (*SMO*, *RAB23*) signaling pathways and genes involved in focal adhesion and cell junctions (e.g. *CD44*, *CDH2*, *PLAU*). Additionally, *COL1A1*, which was not significantly downregulated in qRT-PCR analysis of miR-25-transfected cells (Fig. 1D), and *SMAD4*, a receptor-independent member of the Smad family, showed decreased expression levels in the miR-25 transfected group that were only statistically significant at 24 h but not 48 h post-transfection. The genes upregulated after miR-25 transfection (Fig. 4C) mainly belonged to Wnt/Hedgehog signaling pathway including receptors (*WNT3*, *FZD2 and DVL2–3*), effectors (*GLI1–2*) and signal transducers (*CK1* and *PKC* subunits) as well as downstream cell cycle genes (*CCND2 and CCND3*). Putative regulators of NOTCH signaling, including deltex 2 and 3 (*DTX2* and *DTX3*) and numb-like (*NUMBL*), and transcriptional co/repressors such as *NCOR2*, *SNW1* and *CTBP2* were also upregulated after miR-25 overexpression. Collectively, these data indicate complex regulation of the Notch/Wnt signaling pathways by miR-25 in HSCs, which likely contribute to the pro- or anti-fibrogenic outcomes of HSC activation.

### MiR-25 downregulates ADAM-17 and reduces shedding of NOTCH1

**Figure 5:**
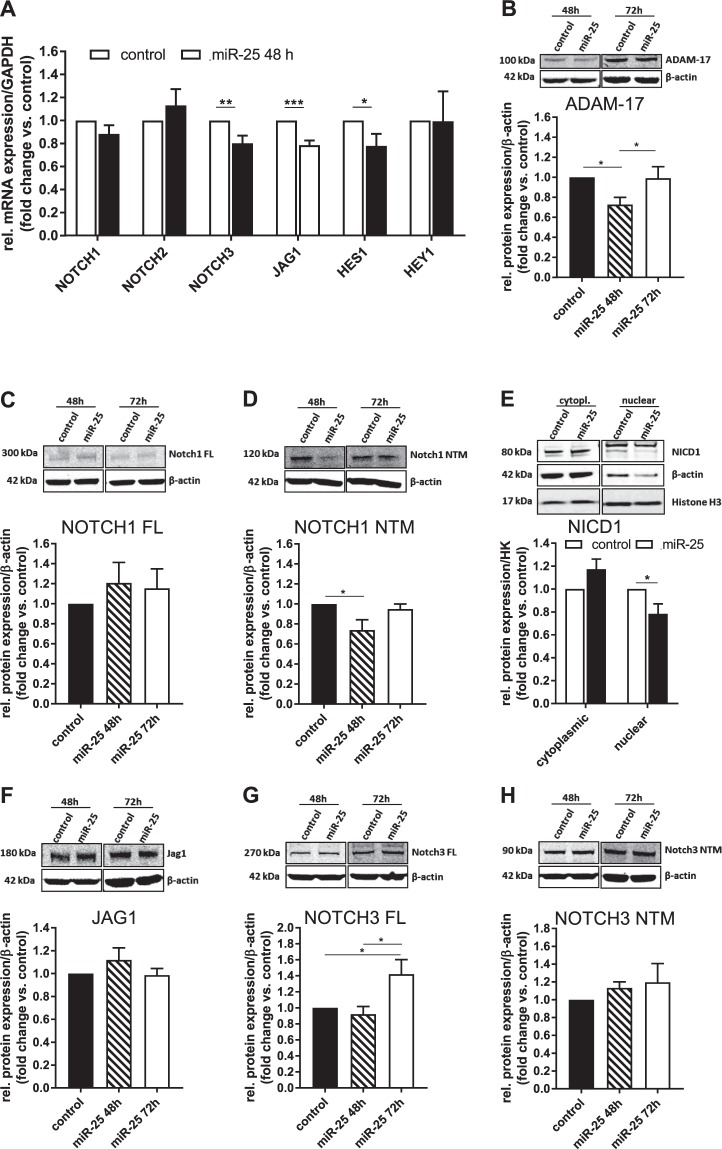
Effect of miR-25 overexpression on Notch signaling in LX-2 cells. (**A**) qRT-PCR analysis of mRNA expression of Notch signaling components in miR-25 transfected LX-2 cells compared to control 48 h after transfection (n = 7–8). Western Blot analysis of (**B**) ADAM-17 (n = 18), (**C**) NOTCH1 full length (FL) (n = 10) and (**D**) N-terminal protein (NTM) (n = 13), in miR-25 overexpressing LX-2 48 and 72 h after transfection. (**E**) Western Blot analysis of NOTCH1 intracellular domain (NICD1) protein expression in nuclear fraction of LX-2 cells 48 h after transfection with miR-25 or control mimics (n = 8). Western Blot analysis of (**F**) Jagged-1 (JAG1) (n = 7), (**G**) NOTCH3 full length (FL) (n = 13) and (**H**) N-terminal protein (NTM) (n = 12) in miR-25 overexpressing LX-2 48 and 72 h after transfection. Cropped Western Blot images originate from the same (target vs. loading control) or different membranes (time points). (cytopl. – cytoplasmatic fraction; *p < 0.05 vs control; **p < 0.01 vs. control; ***p < 0.001 vs. control).

Bansal and colleagues showed that selective blockage of Notch signaling by a γ-secretase inhibitor resulted in reduced activation and contractility of LX-2 cells and ameliorated fibrosis in a murine model of toxin-induced liver fibrosis^8^. Indeed, when we knocked down both Notch activators ADAM-17 and FKBP14 using specific siRNA constructs, TGF-β-induced expression of collagen 1a1, as well as αSMA, was significantly reduced in LX-2 cells (Supplementary Fig. 1). To investigate how miR-25 may dampen pro-fibrogenic signaling in HSCs, we next focused on miR-25-downregulated genes of the Notch pathway. We performed qRT-PCR validation of 6 Notch signaling genes, including miR-25-dependent differentially expressed genes identified by NanoString analysis. We demonstrated the downregulation of both the Notch ligand *JAG1* and its receptor *NOTCH3* at 48 h after transfection (Fig. 5A), in addition to the NOTCH1 sheddase, *ADAM-17* (Fig. 3E). The Notch target gene *HES1* was also decreased by miR-25. To examine whether the effects of miR-25 overexpression on mRNA levels resulted in corresponding protein expression changes we performed Western blot analysis. ADAM-17 protein expression was decreased in LX-2 cells 48 h after transfection, but returned to normal levels at 72 h (Fig. 5B). ADAM-17 is an important sheddase, responsible for the S2 cleavage of the NOTCH1 receptor, making it accessible for S3 cleavage by the γ-secretase complex that finally releases the signaling active Notch1 intracellular domain (NICD1) into the cytoplasm. NICD1 translocates into the cell nucleus to induce expression of Notch-dependent target genes. Whilst *NOTCH1* expression was not affected by miR-25 over-expression at the mRNA level (Fig. 5A), and the expression of full-length (FL) NOTCH1 protein was unchanged (Fig. 5C), the amount of cleaved N-terminal NOTCH1 protein (NTM) was reduced at 48 h post miR-25 transfection (Fig. 5D). The NOTCH1 NTM is the product of S2 cleavage of the NOTCH1 full-length protein and, unlike the NICD1, it still contains the transmembrane component of the receptor. Analysis of the protein abundance of the NICD1 in the nuclear fraction of LX-2 cells revealed a decreased translocation of the active NOTCH1 domain in the nuclei of miR-25-transfected cells at 48 h after transfection (Fig. 5E). miR-25 over-expression did not affect JAG1 (Fig. 5F) or NOTCH3 NTM protein expression (Fig. 5H), but, NOTCH3 full-length protein expression was upregulated at 72 h after miR-25 transfection (Fig. 5G). In summary, miR-25 overexpression inhibited ADAM-17 sheddase expression, which leads to reduced cleavage of the NOTCH1 receptor in LX-2 cells, with subsequent suppression of NICD1 translocation to the nucleus, i.e., inhibition of Notch1 signaling.

### Wnt5a expression is decreased in miR-25 overexpressing LX-2 cells

**Figure 6:**
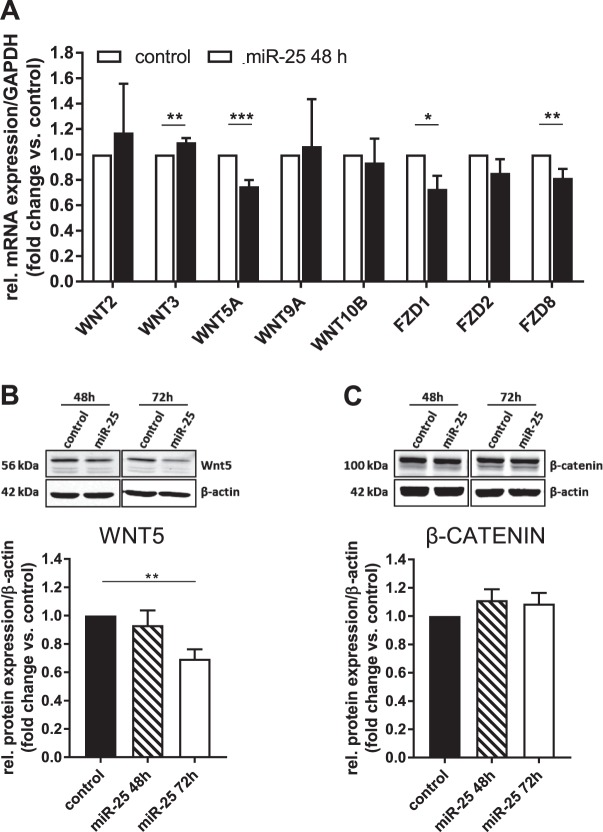
Effect of miR-25 overexpression on Wnt signaling in LX-2 cells. (**A**) qRT-PCR analysis of mRNA expression of Wnt signaling components in miR-25 transfected LX-2 cells compared to control 48 h after transfection (n = 7–8). Western Blot analysis of (**B**) Wnt5 (n = 7) and (**C**) β-catenin (n = 8) in miR-25 overexpressing LX-2 48 and 72 h after transfection. Cropped Western Blot images originate from the same (target vs. loading control) or different membranes (time points) (*p < 0.05 vs control; **p < 0.01 vs. control; ***p < 0.001 vs. control).

It has previously been demonstrated that the NICD can coordinate the cross-talk between several signaling pathways including Wnt^26^. To examine if miR-25 might be involved in regulating the interaction between Notch and Wnt signaling pathways, we further analysed the expression of members of the Wnt signaling pathway in miR-25 overexpressing LX-2 cells. Components of the Wnt signaling pathway (WNT5A, FZD1 and 8) were identified to be downregulated by miR-25 overexpression in qRT-PCR experiments (Fig. 6A), whereas *WNT3* was slightly upregulated 48 h post transfection (Fig. 6A), consistent with the NanoString analysis. Western blot analysis revealed significantly decreased expression of WNT5A, a Wnt ligand involved in either canonical or non-canonical signaling dependent on the receptor it binds to, 72 h after miR-25 transfection (Fig. 6B). There was no effect on β-catenin, a key downstream target of canonical Wnt signaling (Fig. 6C). As the downregulation of WNT5A protein occurs relatively late (72 h) following transfection, this result suggests an indirect inhibition of WNT5A protein expression in LX-2 cells by miR-25 overexpression.

### MiR-25 is upregulated in primary activated murine HSCs and in murine models of hepatic fibrosis

**Figure 7:**
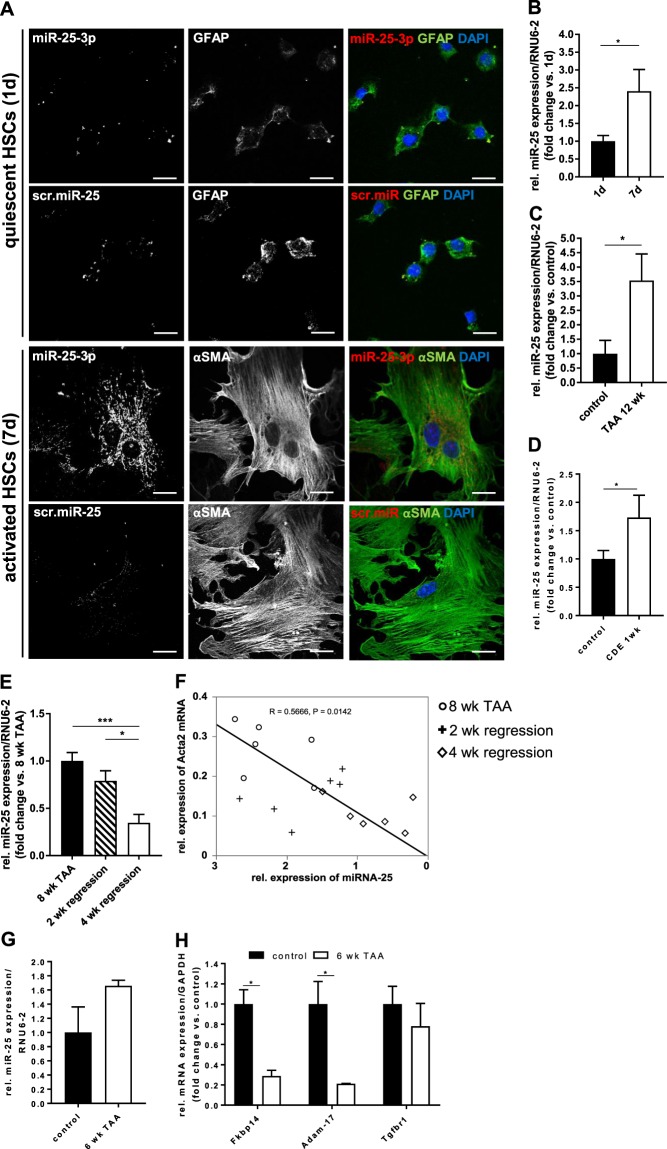
miR-25 expression in murine models of hepatic fibrosis. (**A**) *In situ* hybridization of DIG-labeled miR-25 specific probes (red) in cultured mouse primary HSCs 1 day (quiescent) and 7 day (activated) in culture. Cells were counterstained with HSC-marker GFAP (glial fibrillary protein) or αSMA (green), respectively. A scrambled miR-25 probe was used as negative control (scale bar: 20 µm). (**B**) qRT-PCR expression analysis of miR-25 in primary murine HSCs 1 day (1d) and 7 days (7d) in culture (n = 5). qRT-PCR expression analysis of miR-25 expression in liver tissue of mice (**C**) treated with TAA in the drinking water for 12 weeks (n = 9 per group) or (**D**) fed with a CDE diet for 1 week (n = 7–9 per group). Control animals were kept on standard chow and water. (**E**) qRT-PCR expression analysis of miR-25 in liver tissue from mice treated with TAA for 8 weeks, following fibrosis regression for 2 or 4 weeks after cessation of the toxin (n = 6 per group). (**F**) Analysis of Pearson correlation between liver mRNA expression of the HSC activation marker αSMA (Acta2) and miR-25 in livers of animals regressing from 8 weeks of TAA treatment. The line represents orthogonal regression. qRT-PCR expression analysis of (**G**) miR-25 and (**H**) Fkbp14, Adam-17 and Tgfbr1 mRNA in HSCs isolated from liver tissue of healthy mice (control) or mice treated with TAA for 6 weeks (n = 3–5). (*p < 0.05 vs. control/8 wk TAA; ***p < 0.001 vs. control/8 wk TAA).

To complement the above *in-vitro* studies in the human HSC cell line LX-2, we examined the expression of miR-25 in primary cultures of murine HSCs as well as in mouse models of hepatic fibrosis. Freshly isolated HSCs from healthy mice showed a low abundance of miR-25 expression, which increased during culture-induced HSC activation over 7 days (Fig. 7A,B). *In-situ* hybridization with a miR-25 probe revealed only minor background puncta similar to the scr-miR-25 probe in quiescent HSC (Fig. 7A, upper panel). In contrast, activated HSCs (Fig. 7A, lower panel) showed strong punctate miR-25 expression throughout the cytoplasm, similar to the pattern observed in LX-2 cells (Fig. 1A). qRT-PCR analysis of these cells confirmed the increased expression of miR-25 during the HSC activation process (Fig. 7B). Liver tissue from mice with advanced thioacetamide (TAA)-induced fibrosis (after 12 weeks of TAA administration) showed a marked upregulation of miR-25 expression compared to healthy control animals (Fig. 7C). Increased liver miR-25 expression was also observed after 1-week administration of a pro-fibrogenic choline-deficient ethionine-supplemented (CDE) diet (Fig. 7D). Moreover, miR-25 expression decreased in a time-dependent manner following cessation of TAA treatment (Fig. 7E), which significantly correlated with αSMA [*Acta2*] mRNA expression (R = 0.57, p = 0.0142) in liver tissue of those animals (Fig. 7F). Thus, it appears that miR-25 expression mirrors HSC activation, along with fibrosis progression as well as fibrosis regression in murine models of hepatic fibrosis.

HSCs isolated from livers of mice treated with TAA for 6 weeks revealed an increased expression of miR-25 when compared to HSCs isolated from control mice (Fig. 7G), which confirms the results shown in both culture-induced HSC activation (Fig. 7B) and in whole liver tissue from mouse models of hepatic fibrosis (Fig. 7C,D). Conversely, mRNA expression of miR-25 target genes Fkbp14 and Adam-17 was significantly decreased in HSCs from TAA-treated mice (Fig. 7H). A similar expression pattern was noticable for Tgfbr1 mRNA expression although this did not reach statistical significance (Fig. 7H). Nevertheless, miR-25 expression may not be exclusive to HSCs in the liver as expression analysis demonstrated miR-25 to be present in hepatocyte and macrophage cell lines, although at reduced levels compared to LX-2 cells (Supplementary Fig. 2). Hence, the role of miR-25 in specific liver cell populations and their individual effect on fibrosis development *in vivo* requires further investigation.

## Discussion

Liver fibrosis and its complications such as cirrhosis and hepatocellular carcinoma are a major global health burden with more than 1 million deaths from end stage liver disease in 2010^27^. Despite liver transplantation and antiviral agents, there is no curative anti-fibrotic therapy for the treatment of chronic liver disease available. Independent of the aetiology of chronic liver injury, activated HSCs, along with portal fibroblasts in some hepatic conditions, have been identified as the principal source of excessive extracellular matrix (specifically fibrillar collagen) secretion during fibrosis development^28,29^. Hence, a greater understanding of the mechanisms behind HSC activation and fibrogenesis is vital to identify new anti-fibrotic agents. In this study, we investigated a novel pathway in HSC activation involving miRNA-25-3p.

In recent years, miRNAs including miR-21, miR-29 and miR-122, have been shown to play a role in ‘fine-tuning’ gene expression during processes associated with HSC activation^30–33^. Besides their role in intracellular gene regulation, miRNAs can also be found in extracellular vesicles^16,34^ or bound to extracellular RNA-binding transport proteins^35^ in body fluids and are suggested to function as paracrine or endocrine messengers within the body^15^. Therefore, miRNAs have been widely evaluated as potential biomarkers of various disease states including hepatic fibrosis^19,36,37^. In this regard, we have previously demonstrated a significant decrease of miR-25 in serum of children with cystic fibrosis with associated liver disease, compared to paediatric CF without any sign of liver involvement, suggesting a possible regulatory function of this miRNA in the liver^21^. Here we demonstrate for the first time that miR-25 is expressed in the activated human HSC cell line LX-2 as well as in primary murine HSCs. Fluorescence *in-situ* hybridisation (FISH) experiments revealed a punctate cytoplasmic appearance of miR-25 in both activated primary HSCs and LX-2, whereas in quiescent HSCs the expression was below the detectable level. qRT-PCR analysis of culture-activated primary murine HSCs and HSCs isolated from TAA-treated mice confirmed an activation-dependent expression of miR-25, however, there was no effect of miR-25 overexpression on the expression of generic markers of HSC activation/quiescence such as αSMA, PPARγ, E-cadherin or vimentin. Furthermore, we did not detect any change in cell proliferation in miR-25-overexpressing human LX-2 HSCs, although miR-25 has been shown to promote growth of the tumorigenic hepatocyte cell lines HepG2 and HuH7 *in vitro*^23^ and has been associated with metastasis in esophageal and gastric cancer^38,39^. In contrast, in osteosarcoma, miR-25 has been described to act as a tumour suppressor by inhibiting cell proliferation, migration and invasion^40,41^. These data support a highly cell-, tissue- or context-specific role for miR-25 expression in different disease conditions.

While conventional markers of HSC activation were unaffected by miR-25 overexpression, mRNA levels of TGF-β and its type 1 receptor (TGFβR1) were significantly decreased. Moreover, we demonstrated that miR-25 inhibited TGF-β signaling via Smad2 phosphorylation and the subsequent mRNA induction of collagen 1a1, the principal fibrillar collagen involved in hepatic fibrogenesis, whereas miR-25 inhibition using an antagomir significantly increased TGF-β-induced collagen 1a1 mRNA expression. Interestingly, TGF-β-induced αSMA mRNA expression was unaffected by miR-25 overexpression or inhibition. Different studies suggest that Smad2 and 3 might function via independent pathways in HSCs^42,43^. Consistent with a regulatory role in hepatic fibrogenesis, miR-25 expression was increased in TAA and CDE models of liver fibrosis in mice. MiR-25 has already been shown to regulate TGF-β and collagen expression in extrahepatic organs. Increased TGF-β and collagen 1a1 expression during skin wound healing in mice was associated with reduced expression of miR-25^24^. Furthermore, collagen 1a2 expression was significantly decreased after miR-25 transfection of cardiac fibroblasts^25^ and cardiomyocyte H9c2 cells, whilst miR-25 inhibition reversed collagen downregulation^44^. In renal cancer cells, miR-25 expression was induced by TGF-β treatment and positively correlated with TGF-β expression, while inhibition of miR-25 accelerated TGF-β-induced collagen expression^45^. We did not see an induction of miR-25 expression in LX-2 cells using 5 ng/ml TGF-β (the concentration used in all our experiments), however higher TGF-β concentrations may be required to induce miR-25 expression in LX-2, as they represent already activated HSCs. In summary, these data support a regulatory role for miR-25 during HSC activation, preventing excessive collagen expression in the ongoing presence of TGF-β.

As miR-25 mRNA targets have not previously been investigated in HSCs, we performed pull-down experiments and also analysed the impact of miR-25 overexpression on a panel of 199 mRNAs by NanoString analysis in LX-2 cells to identify biologically and functionally relevant target genes. Analysis and further validation of significantly enriched pull-down targets did not reveal any members of the TGF-β signaling cascade, suggesting the effect of miR-25 on this pathway is indirect. One of the potential limitations of our pull-down methodological approach to identify mRNA targets is that it may not be optimal in detecting mRNAs that are degraded after miRNA binding. Therefore, we performed additional mRNA expression analysis to confirm targets that are strongly downregulated in miR-25- overexpressing LX-2 cells. Thus, we identified several Notch pathway components, including ADAM-17 and FKBP14, as direct miR-25 target genes. NanoString analysis demonstrated miR-25-dependent suppression of ADAM-17, NOTCH1 and NOTCH3 and the downstream transcriptional regulator RBPJ, whilst the inhibitory regulator numb-like (NUMBL)^46^ was upregulated after miR-25 transfection.

Notch signaling is known to be involved in fibrosis development in the liver as well as in other organs^47,48^. Inhibition of Notch signaling via γ-secretase inhibitors or NOTCH3-specific shRNA attenuated liver fibrosis progression and HSC activation in several studies^8,49–51^. In HSCs, Jagged-1 signaling through the NOTCH3 receptor appears to play an important role in myofibroblast transformation, whereas a role for NOTCH1 has not been established^8,52^. Upon ligand binding, Notch receptors are cleaved at the extracellular domain by ADAM (A Disintegrin And Metalloproteinase) sheddases, such as ADAM-17, which facilitates cleavage of the intracellular domain by the γ-secretase complex^48^. In miR-25 overexpressing LX-2 cells, ADAM-17 mRNA and protein were significantly downregulated and cleavage of NOTCH1 receptor, which can be cleaved by ADAM-17 in a ligand-independent manner^53,54^, as well as translocation of the NICD1 to the nucleus, was reduced. Mir-25 overexpression also inhibited NOTCH3 mRNA expression in LX-2 cells, however we did not see an inhibitory effect on protein expression. These data are in line with a study that demonstrated that NOTCH 2 and 3 are not ADAM-17 substrates^55^. Target pull-down and validation experiments also revealed FKBP14 as a target of miR-25 suppression. FKBP14 (FK506 Binding Protein 14) is essential for γ-secretase function and its knock out results in reduction of Notch signaling in drosophila^56^. Moreover, in primary HSCs isolated from fibrotic mouse livers we demonstrated that the mRNA expression of Adam-17 and Fkbp14 was significantly decreased compared to cells isolated from healthy animals. The expression pattern of miR-25 in HSCs isolated from the same mice showed the opposite result, which suggests a potential mechanistic link which would support our *in vitro* observations. Further *in vivo* evidence confirming our proposed miR-25-dependent regulatory mechanism of Notch-regulated TGFβ1-induced collagen expression is required although this was beyond the scope of the current investigation. Together, our data indicate that miR-25 represses NOTCH1-dependent HSC activation at multiple levels, including via downregulation of ADAM-17 and FKBP14. Further mechanistic evaluation is required to determine the precise mechanism involved in this regulation.

There are several suggestions within the literature of bidirectional cross-talk between Notch and TGF-β signaling. Inhibition of Notch activation was shown to suppress TGF-β signaling in kidney fibrosis and TGF-β expression was highly upregulated in NOTCH1 intracellular domain (NICD1) overexpressing tubular endothelial cells^57^. Similarly, in rat peripheral blood mononuclear cells, Notch signaling inhibition resulted in downregulation of TGF-β signaling components and *vice versa*^58^. In a previous study in LX-2 cells, inhibition of Notch signaling by a γ-secretase inhibitor resulted in decreased responsiveness to TGF-β stimulation^8^. It has also been proven that TGF-βR1 is a direct target of NOTCH1 signaling in prostate basal cells^59^. Consistent with these previous studies, we demonstrated reduced expression of Notch pathway components, and reduced NOTCH1 activity (cleavage and nuclear translocation), which was associated with dampened TGF-β-stimulated collagen expression.

Overall, the NanoString analysis highlighted the complex and far-reaching impact of miR-25 overexpression in LX-2 cells. Further validation of selected candidate genes revealed decreased expression of Wnt5a mRNA 48 h, and protein 72 h, after miR-25 transfection. Wnt5a has been shown to potentiate TGF-β signaling in intestinal epithelial cells^60^,and Beljaars and colleagues recently demonstrated that Wnt5a expression is induced by TGF-β, and in turn increases TGF-β-stimulated collagen 1a1, vimentin and fibronectin expression in LX-2 cells^61^. Therefore, we suggest that miR-25 mediated inhibition of autocrine TGF-β signaling may explain the downregulation of Wnt5a in LX-2 cells, which may in turn contribute to the reduced collagen 1α1 expression we observed. Together, our data suggest miR-25 modulates Wnt signaling in HSCs via regulating the expression of specific ligands, receptors and other regulatory molecules.

**Figure 8:**
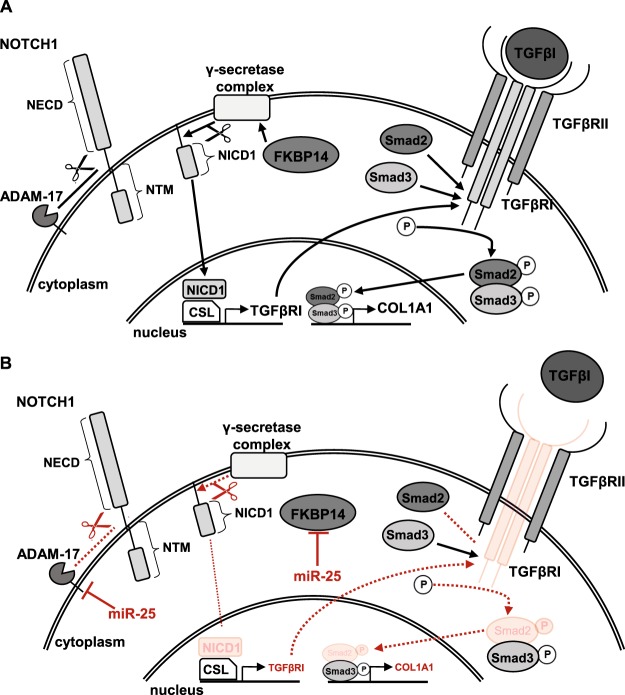
Schematic representation of the putative effect of miR-25 overexpression on TGF-β-induced collagen expression in HSCs. (**A**) In untreated HSCs Notch1 receptor is stepwise cleaved by ADAM-17 sheddase and the γ-secretase complex, which is stabilised by FKBP14 protein, releasing the Notch intracellular domain (NICD1) into the cytoplasm. The NICD1 translocates into the nucleus and induces gene expression of TGF-βR1 by binding to the CSL (CBF1/RBPJ-κ, Suppressor of Hairless, Lag-1) transcription factors. In the cell membrane TGF-β receptor 1 (TGF-βRI) protein dimerises with TGF-βRII to bind TGF-β, resulting in phosphorylation of Smad proteins 2 and 3. The p-Smad2/3 complex then initiates expression of collagen1a1 (COL1A1) in the nucleus. (**B**) HSCs overexpressing miR-25 downregulate expression of ADAM-17 and FKBP14, therefore cleavage of Notch1 receptor is decreased. As a result less NICD1 translocates into the nucleus followed by reduced expression of TGF-βRI in those cells. A reduction of TGF-βRI on the cell surface leads to diminished sensitivity to TGF-β, decreased Smad2 phosphorylation and subsequent COL1A1 expression after TGF-β stimulation.

In conclusion, our findings suggest that miR-25 expression may act as a negative feedback regulator during HSC activation and hepatic fibrogenesis. Being upregulated in activated HSCs, miR-25 downregulates Notch signaling, potentially via multiple mechanisms including ADAM-17 and/or FKBP14-dependent cleavage of NOTCH1 (Fig. 8). As a result, NOTCH1 signaling is diminished, potentially leading to reduced expression of TGF-β and its type 1 receptor, as shown in prostate basal cells^59^. This in turn may reduce TGF-β-induced collagen expression and Wnt5a expression in HSCs. Downregulation of Wnt5a and frizzled receptors and a subsequent reduction in Wnt signaling capacity would also be expected to impact negatively on TGF-β signaling and HSC activation^61^. Reduced Wnt5a secretion may not only affect HSC activation in an autocrine manner, but also regulate fibrogenic processes in neighbouring cells, such as macrophages^62^, in a paracrine manner. Thus we propose that miR-25 acts as a negative regulator of pro-fibrotic stimulation of HSCs during hepatic fibrosis progression. We have presented evidence using both *in vitro* culture-induced activated HSCs as well as HSCs isolated from a murine model of hepatic fibrosis to demonstrate our proposed miR-25-dependent negative feedback regulation driving collagen expression. To provide further evidence of the significance of these novel data, future investigations will be need to assess the impact of HSC-specific overexpression of miR-25 in models of hepatic fibrosis *in vivo*, to fully evaluate the role of miR-25 on the Notch/TGFβ1-signaling pathway cross-talk that drives HSC collagen expression in chronic liver disease.

## Materials and Methods

### Cell culture and miRNA transfection

Primary murine HSCs were isolated from male ex-breeder C57Bl/6 mice as previously described^5,63^ and cultured in Dulbecco’s Modified Eagle Medium (DMEM), 20% fetal calf serum (FCS), 1% glutamin, 1% penicillin/streptomycin at 37 °C, 5% CO_2_ for 0, 1 or 7 days. The human HSC line LX-2 (kindly provided by Prof. Scott L. Friedman, Mount Sinai School of Medicine, NY) was cultured in DMEM, 2% FCS, 1% glutamin, 1% penicillin/streptomycin at 37 °C, 5% CO_2_. Cells were cultured in 6-well plates or 10 cm tissue culture dishes until 80% confluency and transfected with 10 pmol/ml miR-25 or control mimics (MISSION microRNA mimics, Sigma Aldrich, St. Louis, MO, USA) using Effectene transfection reagents (Qiagen, Hilden, Germany) or Lipofectamine LTX (Life Technologies, Carlsbad, CA, USA), according to the manufacturer’s instructions. A non-specific miRNA mimic based upon a *Caenorhabditis elegans* sequence (MISSION microRNA mimics negative control 2; Sigma) served as control. For miR-25 inhibition experiments, LX-2 cells were cultured in 6-well plates until 80% confluency and transfected with 20 pmol/ml miR-25 antagomir (MISSION microRNA inhibitors, Sigma Aldrich) using Lipofectamine LTX transfection reagent (Life Technologies) as recommended by the manufacturer. A non-specific miRNA antagomir based upon a *Caenorhabditis elegans* sequence (MISSION microRNA inhibitor negative control 2; Sigma Aldrich) served as control antagomir.

### Proliferation assays

Cell proliferation was measured using the Incucyte Zoom live content imaging (Essen Bioscience, Ann Arbor, MI, USA). 2 × 10^5^ miR-25- or control-transfected LX-2 cells were seeded (in triplicate) in 12-well plates containing 2 mL of medium and incubated in the Incucyte Zoom machine. Cell growth was measured every 3 hours for up to 7 days and plotted as percentage confluence using the Incucyte analysis software. Alternatively, proliferation was measured by the MTT method, where cells were seeded in 96-well tissue culture plates at a density of 4 × 10^3^ cells per well and transfected with miR-25 or control mimics. After 24 h cells were incubated with MTT (3-(4,5-Dimethylthiazol-2-yl) -2,5-diphenyltetrazolium bromide) reagent (5 mg/ml) for 4 h at 37 °C. Cells were then washed, incubated for 15 min with DMSO and the absorbance was measured at 590 nm (620 nm reference filter) in a spectrophotometer.

### qRT-PCR analysis

For quantitative real-time PCR analysis RNA was isolated from miRNA-transfected LX-2 cells using the RNeasy Kit (Qiagen) or from primary isolated HSCs using the Isolation II RNA Mini Kit (Bioline, Luckenwalde, Germany) as recommended. 1 µg (LX-2) or 100 ng (primary HSCs) of RNA was transcribed into cDNA using SensiFAST^TM^ cDNA synthesis kit (Bioline) followed by qRT-PCR with Platinum SYBR Green (Life Technologies) on a standard protocol (50 °C 2 min, 95 °C 2 min; 40 cycles: 95 °C 15 sec, 60 °C 30 sec). Primers are listed in Supplementary Table 1. Relative expression of mRNA was calculated by *Pfaffl* method^64^. Target gene expression was normalized to the relative expression of the housekeeping gene Glyceraldehyde 3-phosphate dehydrogenase (GAPDH).

### TGF-β stimulation assay

For TGF-β stimulation experiments LX-2 cells were seeded in 6-well plates at a density of 2–3 × 10^5^ cells per well. Cells were serum starved overnight and transfected with miR-25 or control mimics, vs. miR-25 or control antagomirs, respectively, as described above. One day post transfection cells were treated with recombinant human TGF-β (5 ng/ml; Sigma) or an equal amount of phosphate-buffered saline (PBS) as control in serum-free medium. Cells were lysed after 6 h for subsequent protein analysis or after 24 h for RNA isolation.

### Biotin pull-down assay and Illumina microarray hybridization

Biotin-based pull-downs were performed as previously described^65^. Briefly, LX-2 cells were grown in 10 cm tissue culture dishes until >80% confluent. 50 pmol of biotin-labelled miR-25 duplex (5′-CAUUGCACUUGUCUCGGUCUGAAG -Biotin-3′; 3′-GAGCAACGUGAACAGAGACAGACU-5′) was combined with Lipofectamine 2000 (Life Technologies) and transiently transfected into LX-2 cells for 24 h. Cells were then lysed with freshly prepared lysis buffer (10 mM KCl, 1.5 mM MgCl_2_, 10 mM Tris-Cl pH7.5, 5 mM DTT, 0.5% Sigma-IGEPAL, 60 U/mL SUPERase inhibitor, 1x Complete Mini protease inhibitor). Total lysate was used as a control while the remaining lysate was combined with pre-washed and blocked MyOne Streptavidin C1 Dynabeads (Invitrogen, Carlsbad, CA, USA) to capture miRNA duplexes and bound mRNA targets. Captured mRNA (50 ng) was amplified and labelled using the Illumina Total Prep RNA Amplification Kit (Ambion, Carlsbad, CA, USA) as per the manufacturer’s instructions. Samples were hybridized and run on Illumina Human HT-12 chips. Profiling was performed from three independent biological replicates per group.

Array data were exported from Genome Studio (Illumina, San Diego, CA, USA) and analysed as described previously^66^. Briefly, data were normalised using the lumi package^67^ followed by testing for differential expression between control and pull-down samples using the lmFit and eBayes functions in the limma package in R^68^. Probes that met a 5% false discovery rate threshold, had an adjusted p-value <0.05 and a fold-change >2 were considered significantly enriched. Matching transcripts to these probes were deemed putative targets of miR-25. Targets that met the aforementioned criteria were analysed with Ingenuity Pathway Analysis (Qiagen Bioinformatics) for overlap of predicted targets with TargetScan (version 7.2^69^) and miRDB^70^ prediction databases.

### Luciferase reporter assay

MiRNA target binding was validated by Dual Luciferase Assay (Promega, Madison, WI, USA). DNA vectors containing clones of target 3′UTRs (pMiRTarget; Origene, Rockville, MD, USA) were co-transfected with miRNA or control mimics into LX-2 cells following manufacturer’s recommendations. Luciferase activities were measured 48 h after transfection with a bioluminescence plate reader.

### NanoString nCounter assays and analysis

To analyse mRNA expression on the NanoString nCounter platform, LX-2 cells were transiently transfected with miR-25 or control mimics in a 6-well plate as described above. In parallel one well of each plate was transfected with a fluorescein-labelled control miRNA (Bioneer Corporation, Daejeon, South Korea) and transfection efficiency was set as percentage of fluorescein-positive cells from all cells. RNA from miR-25 and control miRNA transfected LX-2 cells was extracted using the RNeasy Kit (Qiagen). The differential expression of 199 genes relating to pluripotency and cell differentiation was investigated using the nCounter Dx Stem Cell Panel, following the manufacturers XT Assay protocol (MAN-10023-11). Briefly, 50 ng of RNA was hybridised to the Reporter and Capture probes at 65 °C for 16 h. The samples were loaded onto the nCounter Prep Station to wash off unbound probes and immobilised on the cartridge for data collection. The target probes were digitally counted using the nCounter Digital Analyser. The data were processed using nSolver Analysis Software version 3.0. The raw counts were corrected using background probes, and scaling was performed using positive probes as per nSolver standard analysis by the manufacturer. Corrected RNA counts for genes in each array were normalised to the average counts in each sample (i.e. normalized counts = N/average of all counts). The ratio of expression of genes after miRNA transfection was calculated against control transfection for each replicate (n = 3 per time point). In order to correct for transfection efficiency, we defined unchanged genes as those with average ratio (across the triplicates) of miR-25/control between 0.98 and 1.02. Genes with miR-25/control ratios <0.98 were defined as downregulated and the ratios were corrected by multiplication with transfection efficiency. Genes with miR-25/control ratios >1.02 were defined as upregulated and the ratios were corrected by dividing by transfection efficiency. The normalized and transfection efficiency-corrected data are shown in Supplementary Data 2. Genes that were expressed in all samples and deregulated by ectopic expression of miR-25 were subjected to pathway analysis and protein-protein interaction (PPI) using STRING (v10)^71^.

### miRNA isolation and relative quantification

Isolation of miRNA from LX-2 cells was performed using an adapted protocol for the RNeasy Kit (Qiagen). For miRNA isolation from liver tissue or primary HSCs samples were homogenized in TRI reagent and RNA was extracted by adding chloroform followed by precipitation with isopropanol. For quantitative analysis 100 ng (primary HSCs) or 2 µg (LX-2 cells, liver tissue) of RNA were transcribed into cDNA using the miScript II RT Kit (Qiagen) according to manufacturer’s instructions. Afterwards real-time PCR analysis was performed using the miScript SYBR^®^ Green PCR Kit and appropriate Primer Assays (Qiagen) as recommended in the manufacturer’s protocol. Relative expression of miRNA was analysed using the 2^ΔCT^ method normalizing all samples to the relative expression of the housekeeping gene RNU6-2.

### Western Blot analysis

Cells were lysed in RIPA buffer (2% NP-40, 0.05% Na-deoxycholate, 0.1% sodium dodecyl sulfate (SDS), 1x protease and phosphatase inhibitor in PBS) and 10 or 20 µg protein per sample were separated by SDS polyacrylamide gel electrophoresis (SDS-PAGE) and transferred to a low-fluorescent PVDF membrane (Merck Millipore, Burlington, MA, USA). After blocking with Odyssey® TBS blocking buffer (Li-Cor Biosciences, Lincoln, NE, USA) for 1 h at room temperature membranes were incubated with the following primary antibodies: anti-Wnt5 (1:2000; ProSci Inc., Poway, CA, USA), anti-ADAM-17 (1:1000; Abcam, Cambridge, UK) anti-Smad2, anti-p-Smad2, anti-Smad3, anti-p-Smad3, anti-β-catenin, anti-Notch1, anti-Notch3, anti-Histone H3 (all 1:1000; Cell Signaling Technologies, Danvers, MA, USA), anti-activated Notch1 (1:1000; Abcam) and anti-β-actin (1:10,000; Sigma) for 4 °C overnight. Signal detection was obtained by incubating the membranes with the corresponding secondary antibodies anti-mouse-IRDye680 (1:20,000) as well as anti-rabbit-IRDye800 (1:20,000; both Li-Cor) for 1 h at room temperature and subsequently scanning with an Odyssey® CLX infrared imaging system (Li-Cor). Relative sample quantification was performed with Image Studio Lite Software version 5.2.5 (Li-Cor) and images were prepared for display with ImageJ software 1.51j8 (NIH, USA). All full-length blots are provided in Supplementary Information (Supplementary Fig. 3).

### Fluorescence *in situ* hybridization (FISH)

LX-2 cells and primary HSCS were seeded on glass cover slips and cultured as described above. Cells were fixed with cold methanol for 2 min. Cover slips were incubated with Digoxigenin-labelled DNA-oligo probes (miR-25: 5′-/5DigN/TCAGACCGAGACAAGTGCAATG/3DigN/-3′; scrambled: 5′-/5DigN/GATAGGTA GCGACATACACCGA/3DigN/-3′; 1 pmol/µl in hybridization buffer [40 mM HEPES, 0.4 M NaCl, 2 M Urea, 1 mM EDTA]) at 37 °C overnight in a humidified chamber. After blocking the cells in 1% bovine serum albumin in PBS, probes were detected by using an anti-Digoxigenin antibody (1:500, 4 °C, overnight; Vector Laboratories, Burlingame, CA, USA) and an appropriate anti-goat-Alexa-594 secondary antibody (1:1000, 1 h at room temperature; Life Technologies). In parallel, primary HSCs were stained for HSC marker glial fibrillary protein (GFAP) or alpha smooth muscle actin (αSMA) with anti-GFAP (1:300; Cell Signaling) or anti-αSMA (1:1000; Sigma) primary antibodies and subsequent anti-mouse-Alexa488 secondary antibody (1:1000, Life Technologies), all for 1 h at room temperature. Finally, cover slips were mounted with fluorescence mounting medium containing DAPI and visualised with a confocal laser scanning microscope.

### Animal models of liver fibrosis

All animal experiments performed were approved and monitored by the University of Queensland (MED/PAH/156/13/PAHRF/NHMRC, UQDI/571/12/NHMRC/AIDRCC) and Curtin University (AEC_2013_36) Animal Ethics Committees. All procedures were conducted in accordance with the “Australian code of practice for the care and use of animals for scientific purposes” (Australian National Health & Medical Research Council). To induce the TAA cytotoxic liver fibrosis model, 6- to 9-week-old mice were exposed to 300 mg/L thioacetamide (TAA, Sigma) in their drinking water over a period of 6 or 12 weeks^72,73^, or 8 weeks with a subsequent regression phase of 2 or 4 weeks. In a second model, fibrosis was induced by feeding mice with a modified choline-deficient ethionine-supplemented diet for 1 week^74,75^. The diet was custom-made by MP Biosciences (Santa Ana, USA) in a 70:30 (choline-deficient vs. choline-sufficient) composition. At the individual time points, mice were sacrificed and liver samples were homogenised in TRI reagent (Invitrogen) and stored at −80 °C for RNA isolation.

### Statistical analysis

All data represent at least 3 independent experiments and are represented as mean ± standard error of the mean. Statistical analyses were performed using GraphPad Prism version 7.02 (GraphPad Software). Depending on data composition significant differences between groups were tested using either one sample t-test or Wilcoxon signed rank test as well as One- and Two-way ANOVA with Tukey post hoc multiple comparison test. p < 0.05 was defined statistical significant.

## Supplementary information

Supplementary Information

Supplementary Dataset 1

Supplementary Dataset 2

## Data Availability

All data generated or analysed during this study are included in this published article (and its Supplementary Information Files). Additionally, the microarray and NanoString data are available from the Gene Expression Omnibus (GEO) under accession numbers GSE119019 (Illumina microarray) and GSE119022 (NanoString).
