# Supplementary material for: Overexpression of miRNA-25-3p inhibits Notch1 signaling and TGF-β-induced collagen expression in hepatic stellate cells
Source: Sci Rep. 2019 Jun 12;9:8541. doi: 10.1038/s41598-019-44865-1 (PMC6561916; doi:10.1038/s41598-019-44865-1)
Supplement: Supplementary file 1 — Supplementary Information [file 41598_2019_44865_MOESM1_ESM.pdf]

Overexpression of miRNA-25-3p inhibits Notch1 signaling and TGF-β-induced collagen expression in hepatic stellate cells

Berit Genz[1,2,3], Miranda A. Coleman[1], Katharine M. Irvine[2,3], Jamie R. Kutasovic[3,4], Mariska Miranda[4], Francis D. Gratte[5,6], Janina E. E. Tirnitz-Parker[5], John. K. Olynyk[7,8], Diego A. Calvopina[1], Anna Weis[1], Nicole Cloonan[9], Harley Robinson[10], Michelle M. Hill[10], Fares Al-Ejeh[4] and Grant A. Ramm[1,3,*]

[1]Hepatic Fibrosis Group, QIMR Berghofer Medical Research Institute, Brisbane, Queensland, Australia.

[2]Mater Research, Translational Research Institute, Brisbane, Queensland, Australia

[3]Faculty of Medicine, The University of Queensland, Brisbane, Queensland, Australia

[4]Personalised Medicine Team, QIMR-Berghofer Medical Research Institute, Brisbane, Queensland, Australia

[5]School of Pharmacy and Biomedical Sciences, Curtin Health Innovation Research Institute, Curtin University, Bentley, WA, Australia

[6]School of Veterinary and Life Sciences, Murdoch University, Perth, Western Australia, Australia

[7]Department of Gastroenterology & Hepatology, Fiona Stanley Fremantle Hospital Group, Murdoch, Western Australia, Australia

[8]School of Medical and Health Sciences, Edith Cowan University, Joondalup, Western Australia, Australia

[9]Genomic Biology Lab, QIMR-Berghofer Medical Research Institute, Brisbane, Queensland, Australia

[10]Precision & Systems Biomedicine, QIMR-Berghofer Medical Research Institute, Brisbane, Queensland, Australia

** corresponding author:* Professor Grant A. Ramm, BSc, BSc(Hons), PhD, FAASLD
Hepatic Fibrosis Group, QIMR Berghofer MRI
300 Herston Road, Herston QLD 4006, Australia.
grant.ramm@qimrberghofer.edu.au

Supplementary Table S1. Primer sequences used for qRT-PCR.

Human genes	Primer	Sequence (5' to 3')
**ACTA2**	forward	CTATCAGGGGGCACCACTATGTA
	reverse	GCTCCGGAGGGGCAATGA
**ADAM-17**	forward	CCATGAAGTGTTCCGATAGAT
	reverse	ACCTGAAGAGCTTGTTCATCG
**COL1A1**	forward	AAGCCGAATTCCTGGTCTGG
	reverse	CGATGGCTGCACGAGTCACA
**CDH1**	forward	CTGGTTCAGATCAAATCCAACA
	reverse	CTTCAGCCATCCTGTTTCTCTT
**FKBP14**	forward	ACGGCTCCTTATTTCACTCCAC
	reverse	ACACATTCCTTTCAAGCCCT
**FZD1**	forward	TGCCCTCCTACCTCAACTACCA
	reverse	GCACTGACCAAATGCCAATCCA
**FZD2**	forward	CCCGACTTCACGGTCTACAT
	reverse	CTGTTGGTGAGGCGAGTGTA
**FZD8**	forward	GACACTTGATGGGCTGAGGT
	reverse	CAAATCTCGGGTTCTGGAAA
**GHAPDH**	forward	GTCCACCACCCTGTTGCTGTAG
	reverse	GACACCCACTCCTCCACCTTTGA
**HES1**	forward	CTGAGCACAGACCCAAGTGT
	reverse	GAGTGCGCACCTCGGTATTA
**HEY1**	forward	GCGTGGGAAAGGATGGTTGAG
	reverse	CTCTCGGCTGCTTGCGTTC
**JAG1**	forward	GAGCTATTTGCCGACAAGGC
	reverse	GGAGTTTGCAAGACCCATGC
**LEP**	forward	GACTTTTTGGATGGGCACAG
	reverse	GTAGGAATCGCAGCGCC
**NOTCH1**	forward	CCACCTCGTCTCTCCCACCT
	reverse	ACAGCCACTCGCATTGACCATT
**NOTCH2**	forward	TGCCAAGGGTAGTAGGAGGAAGAA
	reverse	TGGAGAGGATGTGGTGTCGGAA
**NOTCH2NL**	forward	ACCCTCGCCTTGTGTCAATGG
	reverse	CTGTTCCTCTTCTCACTGTTTCTGG
**NOTCH3**	forward	ACACCAATGCCCAGGACCAC

	reverse	ATCAGTGCCGTTGAGCCATCTG
**PPARG**	forward<br>reverse	GGCTTCATGACAAGGGAGTTTC<br>AACTCAAACTTGGGCTCCATAAAG
**PTGR2**	forward<br>reverse	GAACAGCTCCGTGAATCATG<br>GTGGCTGTTCTCATTCATCTG
**TGF-β1**	forward<br>reverse	TGGAAACCCACAACGAAATC<br>GGGTTCAGGTACCGCTTCTC
**TGFbR1**	forward<br>reverse	GCCTTGGTCCTGTGGAACTG<br>GGTCCTCTTCATTTGGCACTCG
**VIM**	forward<br>reverse	GTGAATACCAAGACCTGCTCAA<br>AGGGAGGAAAAGTTTGGAAGAG
**WNT2**	forward<br>reverse	AGCAGGCGTCACCATCCAC<br>GGGCTTCCGTTGAGATAAAGGC
**WNT3**	forward<br>reverse	AAGTTAGACAAAGGGTCCGTGAGG<br>TGGAGAGGAAGCAAAGGGATGGTT
**WNT5A**	forward<br>reverse	CAACTGGCAGGACTTTCTCA<br>TTCTTTGATGCCTGTCTTCG
**WNT9A**	forward<br>reverse	ACCCGACCTGGAGAACCGT<br>TCCTTGACGAACTTGCTGCTG
**WNT10B**	forward<br>reverse	GCAACAAGACCAGCCGCC<br>TCACACAGCACATAGCAGCACC

**Murine genes**	**Primer**	**Sequence (5' to 3')**
**Acta2**	forward<br>reverse	ACTACTGCCGAGCGTGAGAT<br>CCAATGAAAGATGGCTGGAA
**Adam-17**	forward<br>reverse	CAGAAGAAGTGCCAGGAGGCTA<br>GTTGTCAGTGTCAACGCATGC
**Fkbp14**	forward<br>reverse	TGTGGAACGCTATCTTGGCA<br>GCCTTCGTAGTGGACCAACA
**Gapdh**	forward<br>reverse	AACTTTGGCATTGTGGAAGG<br>GGATGCAGGGATGATGTTCT
**Tgfbr1**		AAAAGCAGTCAGCTGGCCTT<br>ATGACAGTGCGGTTATGGCA

SUPPLEMENTARY MATERIAL AND METHODS

Cell culture. IHH cells (human immortalised hepatocytes; kindly provided by Prof. Didier Trono, Ecole Polytechnique Fédérale de Lausanne, Lausanne, Switzerland) were cultured in Dulbecco's Modified Eagle's Medium (DMEM) / Nutrient Mixture F-12 Ham (Sigma-Aldrich), supplemented with 10% foetal bovine serum (FBS, Gibco by Thermo Fisher Scientific, Carlsbad, California, United States), 1% L-Glutamine (Life Technologies, Carlsbad, California, United States) and 1x Insulin-transferrin-sodium selenite media supplement (Sigma-Aldrich) containing 5 mg/L insulin, 5 mg/L transferrin and 5 µg/L selenium. Huh7 (human hepatoblastoma cells) and RAW 264.7 (murine monocytes-derived macrophages) cell lines were cultured in DMEM containing 4500 mg/L glucose (Sigma-Aldrich) supplemented with 10% FBS (Gibco by Thermo Fisher Scientific) and 1% L-Glutamine (Life Technologies). All cells were cultured at 37°C in a 5% CO_2 containing humidified atmosphere.

siRNA transfection. LX-2 cells were cultured in 6-well plates until 80% confluency and transfected with 25 nmol/ml ADAM-17, FKBP14 or Firefly Luciferase (FLUC) siRNA (MISSION esiRNA [endoribonuclease-prepared siRNAs], Sigma Aldrich, St. Louis, MO, USA) using Lipofectamine LTX (Life Technologies, Carlsbad, CA, USA) according to the manufacturer's instructions. FLUC siRNA served as control. Cells were cultured for 48 h before harvesting for RNA isolation.

Suppl. Figure 1

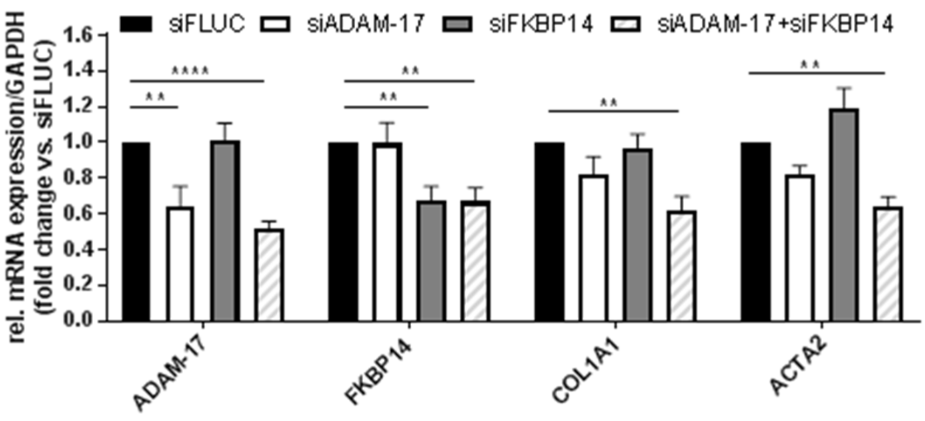

Suppl. Figure 1: Effect of siADAM-17 and siFKBP14 (alone and in combination) on the mRNA expression of ADAM-17, FKBP14, COL1A1 and ACTA2 in the presence of 5 ng/ml TGFβ in LX-2 cells. (*p < 0.05, **p < 0.01, ****p < 0.0001, 2way-ANOVA, n=6). siFLUC – control siRNA against firefly luciferase

Suppl. Figure 2

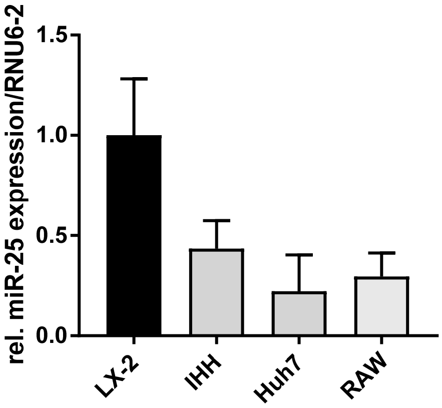

Suppl. Figure 2: qPCR analysis of miR-25 expression in different liver cell lines (n=3). LX-2 (human HSCs), IHH (human immortalised hepatocytes), Huh7 (human hepatoblastoma cells), RAW 264.7 (murine monocytes-derived macrophages)

Suppl. Figure 3

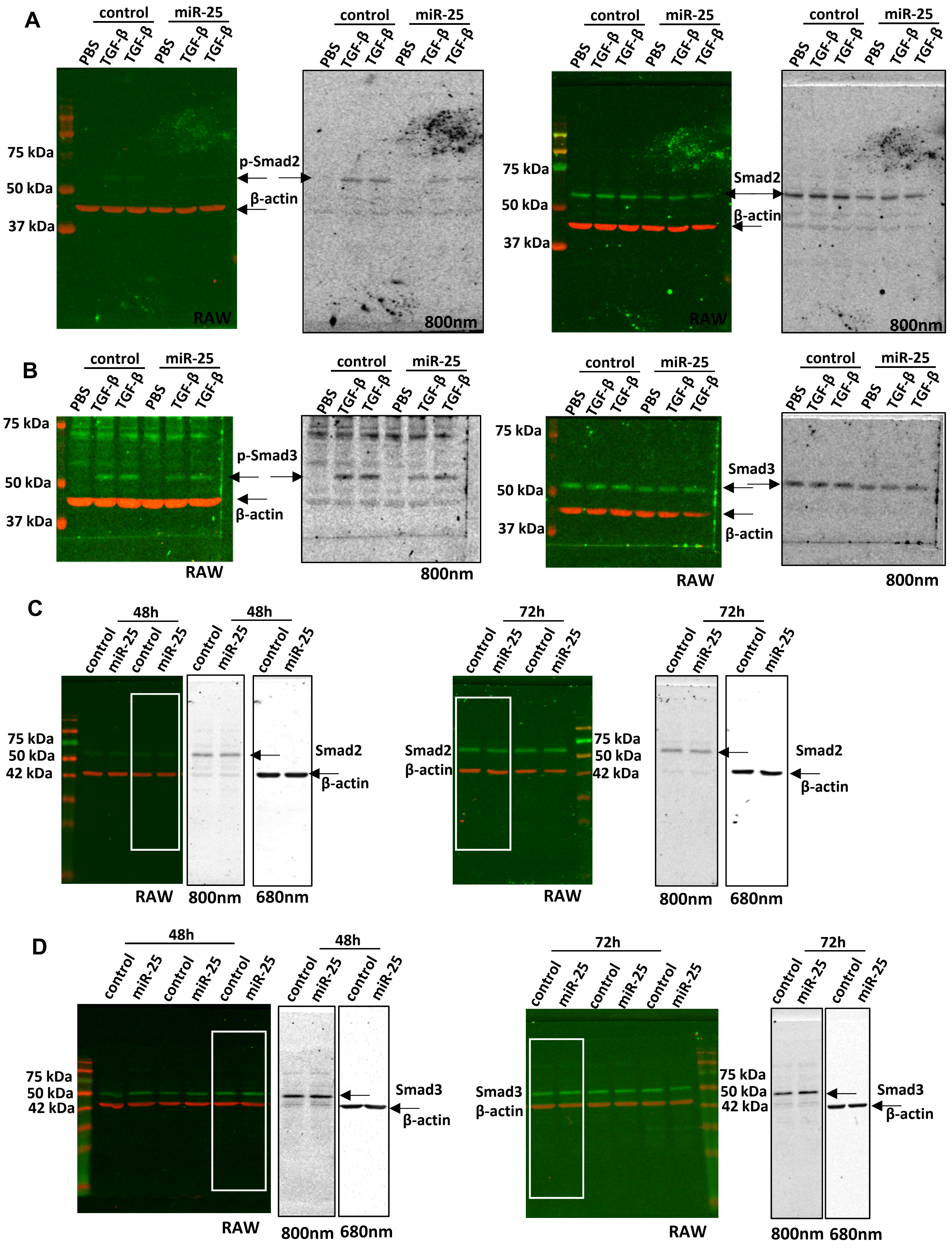

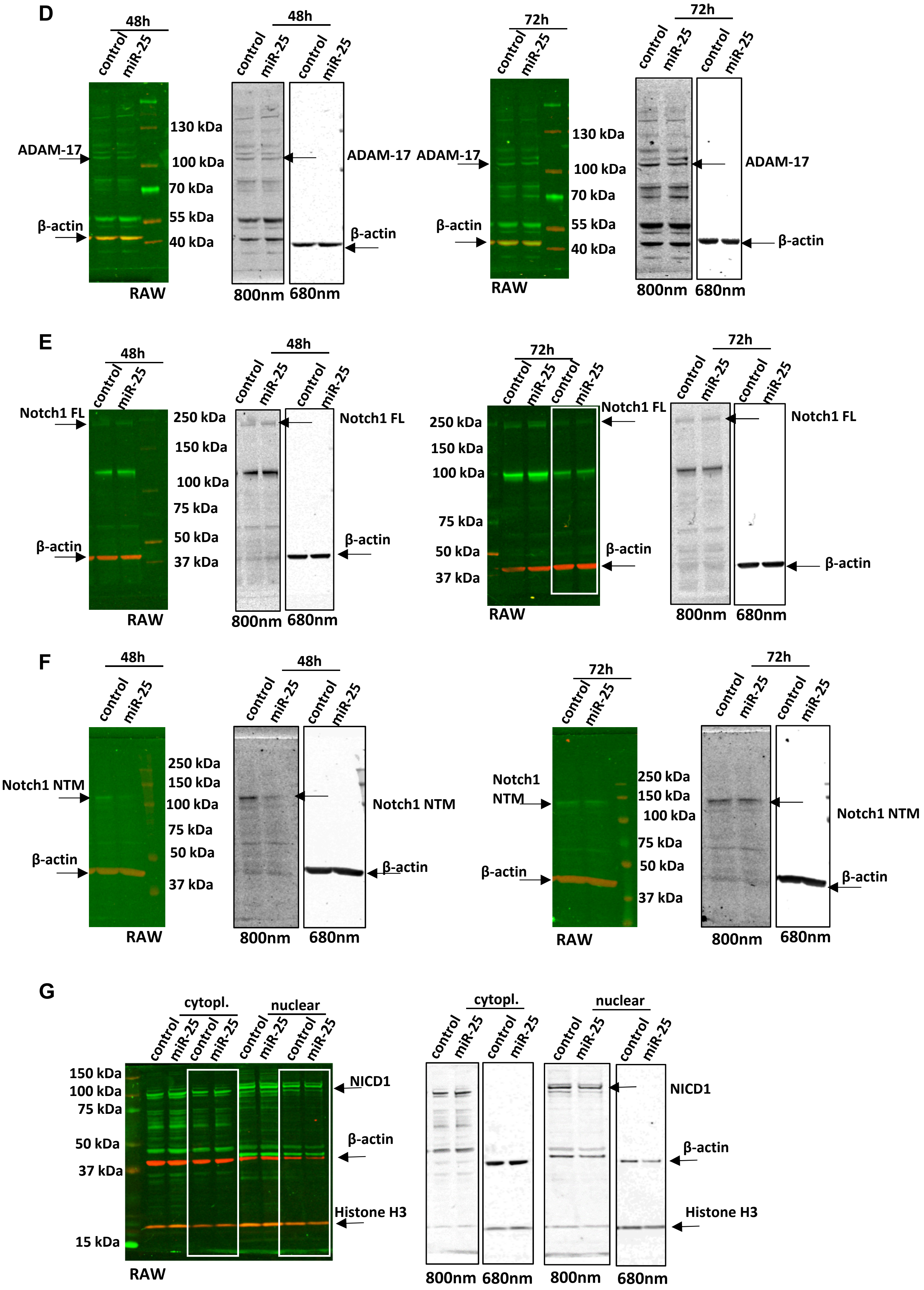
D
48h
control
miR-25
ADAM-17
β-actin
130 kDa
100 kDa
70 kDa
55 kDa
40 kDa
RAW
800nm
680nm
72h
E
Notch1 FL
250 kDa
150 kDa
100 kDa
75 kDa
50 kDa
37 kDa
F
Notch1 NTM
G
cytopl.
nuclear
NICD1
Histone H3
15 kDa

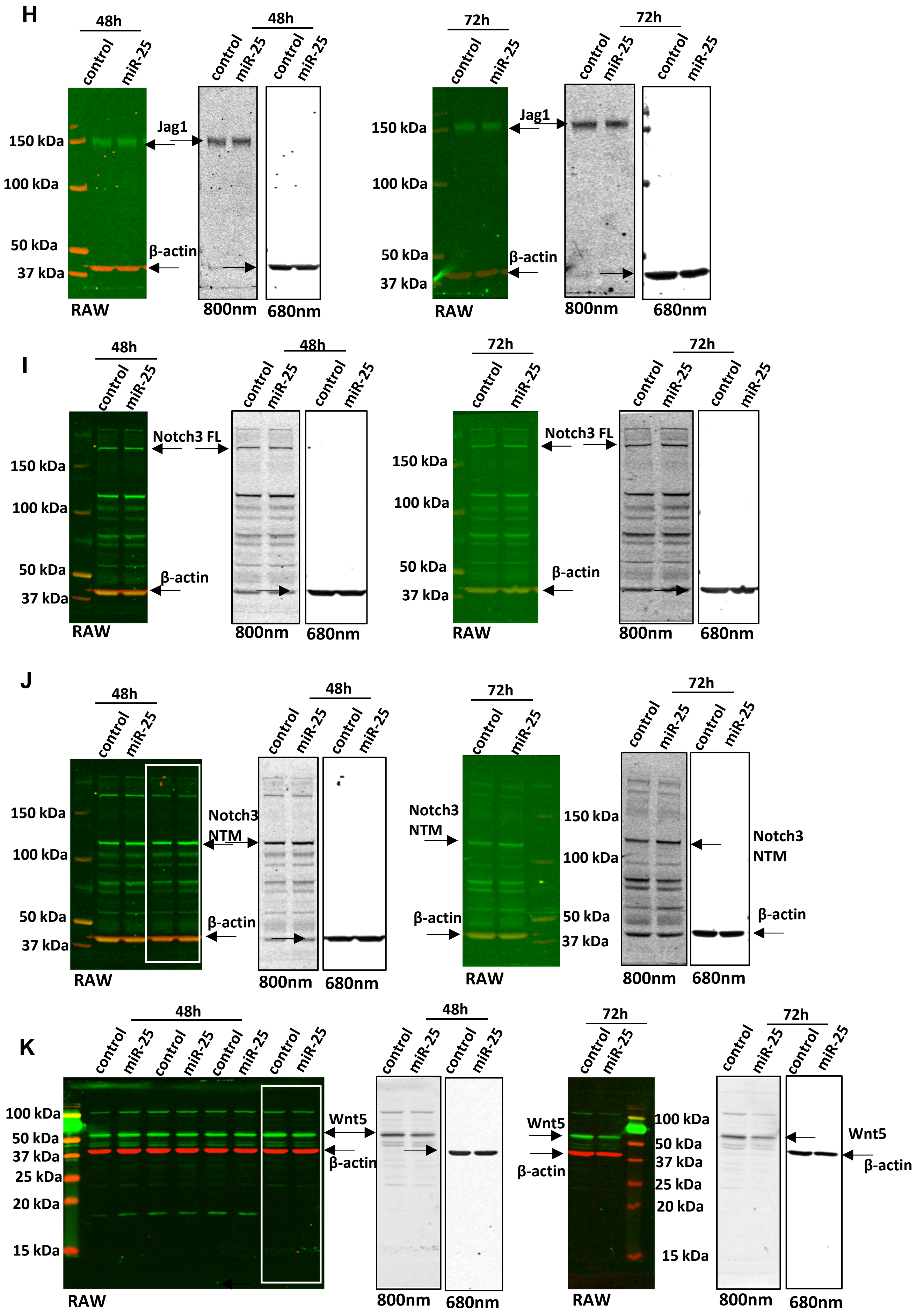

H
48h
control
miR-25
150 kDa
100 kDa
50 kDa
37 kDa
Jag1
β-actin
RAW
800nm
680nm
72h
I
Notch3 FL
J
Notch3 NTM
K
Wnt5
25 kDa
20 kDa
15 kDa

L

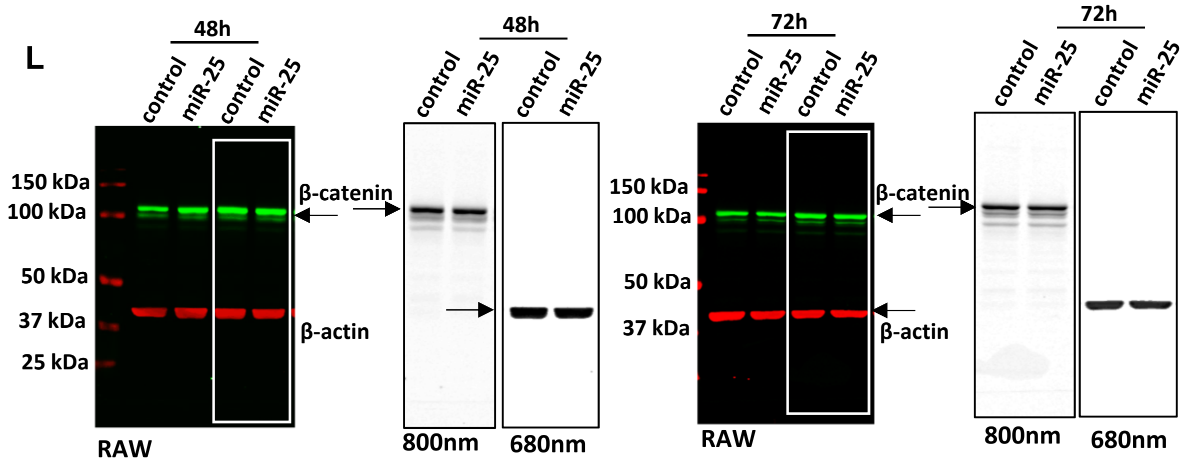
48h
control
miR-25
control
miR-25
150 kDa
100 kDa
50 kDa
37 kDa
25 kDa
β-catenin
β-actin
RAW
48h
control
miR-25
control
miR-25
800nm
680nm
72h
control
miR-25
control
miR-25
150 kDa
100 kDa
50 kDa
37 kDa
β-catenin
β-actin
RAW
72h
control
miR-25
control
miR-25
800nm
680nm
